# Progression of Hepatic Adenoma to Carcinoma in *Ogg1* Mutant Mice Induced by Phenobarbital

**DOI:** 10.1155/2017/8541064

**Published:** 2017-07-13

**Authors:** Anna Kakehashi, Naomi Ishii, Takahiro Okuno, Masaki Fujioka, Min Gi, Shoji Fukushima, Hideki Wanibuchi

**Affiliations:** Department of Molecular Pathology, Osaka City University Graduate School of Medicine, Abeno-Ku, Asahi-Machi 1-4-3, Osaka 545-8585, Japan

## Abstract

The carcinogenic potential of phenobarbital (PB) was assessed in a mouse line carrying a mutant *Mmh* allele of the *Mmh/Ogg1* gene encoding the enzyme oxoguanine DNA glycosylase (Ogg1) responsible for the repair of 8-hydroxy-2′-deoxyguanosine (8-OHdG). *Mmh* homozygous mutant (*Ogg1*^−/−^) and wild-type (*Ogg1*^+/+^) male and female, 10-week-old, mice were treated with 500 ppm PB in diet for 78 weeks. Hepatocellular carcinomas (HCCs) were found in PB-treated *Ogg1*^−/−^ mice, while *Ogg1*^+/+^ animals developed only hepatocellular adenomas (HCAs) at the same rate. This was coordinated with PB-induced significant elevation of 8-OHdG formation in DNA and cell proliferation in adjacent liver of *Ogg1*^−/−^ mice. Proteome analysis predicted activation of transcriptional factor Nrf2 in the livers and HCAs of PB-administered *Ogg1*^+/+^ mice; however, its activation was insufficient or absent in the livers and HCCs of *Ogg1*^−/−^ mice, respectively. Significant elevation of phase I and II metabolizing enzymes was demonstrated in both *Ogg1*^−/−^ and *Ogg1*^+/+^ animals. Treatment of *Ogg1*^−/−^ mice with PB resulted in significant elevation of cell proliferation in the liver. These results indicate that PB induced progression from HCA to HCC in *Ogg1*^−/−^ mice, due to persistent accumulation of DNA oxidative base modifications and suppression of Nrf2-mediated oxidative stress response, resulting in significant elevation of cell proliferation.

## 1. Introduction

Elevation of oxidant byproducts of intercellular metabolism induces DNA damage which is the key factor in cancer and aging. Furthermore, the age-dependent changes in the efficacy and the rate of DNA repair in mammals regulate the susceptibility to endogenous or exogenous carcinogens [1]. Reactive oxygen species (ROS) induce a variety of lesions in DNA, including oxidized bases, DNA strand breaks, and abasic sites, but most frequently attack the 8 position of guanine producing an oxidative DNA damage marker, 8-hydroxy-2′-deoxyguanosine (8-OHdG) [2]. The reason of increased frequency of spontaneous GC → TA transversion mutations detected in repair-deficient bacterial and yeast cells was reported to be elevated levels of 8-OHdG which is strongly mutagenic, being able to mispair with A residues [3, 4].

In previous studies, increase of repair-resistant oxidatively induced clustered DNA lesions in the human oxoguanine glycosylase 1 (*Ogg1*) gene and other repair enzymes were observed in patients with liver, ovary, kidney, breast, and colon cancers [5]. Furthermore, loss of heterozygosity (LOH) in *Ogg1* was suggested to play an important role in the development of human hepatocellular carcinomas (HCC), being an early event in hepatocarcinogenesis [6]. In addition, polymorphisms of DNA repair genes including *Ogg1* were associated with the overall survival of HCC patients with chronic HBV infection [7]. Decreased expression of Ogg1 and mitochondrial Ogg1 (mtOGG1) were reported with human HCC tissues and SNU (Seoul National University) human hepatoma cell lines, respectively [8].

Three enzymes from *Saccharomyces cerevisiae* and various bacteria are known to prevent spontaneous mutagenesis induced by the 8-OHdG [9]. One of them, the Fpg (MutM) DNA glycosylase-AP lyase, removes the oxidized base from G:C base pairs in duplex DNA. The second one, MutY DNA glycosylase, specifically excises adenine misincorporated during replication, resulting in G-T transversion mutation. The third enzyme, MutT, is a GTPase preventing incorporation of G opposite misincorporated A into nascent DNA by hydrolyzing the excess of dGTP. In addition, both mammalian and yeast cells use a distinct DNA glycosylase, the product of the *Ogg1* gene, to excise nucleotides from DNA. It was reported that cloned human and mouse cDNAs encode distinct nuclear and mitochondrial forms of the enzyme generated by alternative RNA splicing [10–14].

In previous studies, homologues of MutY (MYH) and MutT (MTH) have been identified in mammalian cells [15, 16]. Moreover, a mammalian homologue of glycosylase/apurinic, apyrimidinic lyase (AP lyase; MutM homologue, MMH) has been also identified and cloned [17, 18]. Previously, *Myh* and *Ogg1* knockout mice were demonstrated to develop spontaneously lymphomas and lung and ovary tumors [15, 19]. However, it still remains unclear how the deletion of these enzymes may affect the susceptibility of animals to chemical carcinogens.

*Mmh/Ogg1* mutant mice used in our study are characterized by physically normal appearance, but lack nicking activity in liver extracts for substrate DNA containing 8-OHdG. As compared to the wild-type or heterozygous mice, in the tissues of homozygous mice at 9 and 14 weeks of age, 8-OHdG levels exhibit 3- and 7-fold elevation, respectively [20]. Furthermore, mutation frequency was substantially elevated bearing transgenic *gpt* genes *Mmh/Ogg1* mice [20]. It has been previously reported that administration of potassium bromate (KBrO_3_) to *Ogg1*-deficient mice resulted in a tremendous increase (~70-fold) of kidney DNA, with consequent GC → TA transversions and deletions [10]. In addition, 3.5-fold increase in mutation frequency during liver regeneration following the partial hepatectomy was observed in *Ogg1* mutant mice administered KBrO_3_ [21]. Moreover, dimethylarsinic acid (DMA^V^) was reported to exert carcinogenicity in the lungs of *Ogg1* mutant mice [22].

Phenobarbital (PB), an anticonvulsant and a sedative, used as an antiepilepsy drug in humans, is also a nongenotoxic carcinogen and a well-known promoter of hepatocarcinogenesis in vivo and in vitro [23–26]. The promoting effect of PB at a high dose on hepatocarcinogenesis in rodents has been extensively studied, but reasons for its carcinogenic action have yet to be unequivocally clarified. Increased reactive oxygen species (ROS) generation due to the activity of detoxifying enzymes and formation of 8-OHdG are suggested to be possible mechanisms by which PB may exert carcinogenicity [27]. Chronic PB application is known to induce hepatocellular adenomas (HCAs) but not hepatocellular carcinomas (HCCs) in C57BL/6J, B6D2F1, yellow Avy/A, and agouti A/a mice [28, 29], while in D2B6F1 mice, it was reported to induce hepatoblastomas [30]. Differences in the promoting effects of PB between C57BL/6J and DBA mice appeared to correlate with differences in the metabolism/detoxification of this drug [31].

To address the question, how the deletion of *Ogg1* gene may affect the susceptibility of animals to chemical carcinogens, the present study investigated the carcinogenic potential of nongenotoxic carcinogen PB in the *Mmh/Ogg1* homozygous mutant mice of C57BL/6J background. At the end of the treatment period, multiorgan histopathological, immunohistochemical, biochemical, and proteome analyses were performed focusing on alterations of cell proliferation, apoptosis, formation of oxidative DNA modifications, and protein expression changes in the mouse liver induced by PB.

## 2. Materials and Methods

### 2.1. Chemicals

PB sodium salt (CAS number 57-30-7) (purity ≥ 98%) was purchased from Wako Pure Chemical Industries, Ltd. (Osaka, Japan). Other reagents were from Wako or Sigma.

### 2.2. Maintenance of Mice

The experimental procedures or the present investigation was approved by the Ethics Committee of the Institutional Animal Care and Use Committee of Osaka City University Graduate School of Medicine, Osaka, Japan (approval number 15011), and performed accordingly to the guidelines set by the National Institute of Health and Public Health Service Policy on the Humane Use and Care of Laboratory Animals. *Mmh/Ogg1* mutant (*Ogg1*^−/−^) and wild-type (*Ogg1*^+/+^) C57BL/6J mice of both sexes were bred (approval number 597) in an animal facility with lighting supplied with a 12 h light/dark, at a constant temperature of 22 ± 1°C, relative humidity of 44 ± 5%, and given free access to tap water and food ad libitum. Mice were housed in a SPF zone of our animal house during the experiment, where conditions, such as sterilization and autoclaving procedures, handling sterilized reagents, and disposable plastic ware, are strictly controlled. *Ogg1*^−/−^ and *Ogg1*^+/+^ mice were divided by stratified randomization into 4 body weight-matched groups, comprising male and female animals and housed in plastic cages containing wood chips.

The general behavior, moribund state, or possible signs of toxicity were checked in all mice once a day. Animals body weights and water and food consumptions were measured every week for the first 12 weeks and every 4 weeks after that up to week 78. The signs for euthanization were no response to stimuli or the comatose condition, dyspnea, hypothermia, prostration, body weight loss, severe change of heart rate, or physical appearance. Animals with detected body weight loss were checked more precisely for other signs of sickness.

### 2.3. Experimental Design

*Ogg1* ^−/−^ and *Ogg1*^+/+^ male and female mice were administered a MF pellet diet (Oriental Yeast Co., Tokyo, Japan) containing PB at a dose of 0 (control) or 500 ppm for 4 (10 mice/group) or 78 (20 mice/group) weeks, respectively.

In the 4-week experiment (Exp. 1), mice were euthanized under the isoflurane, and livers immediately excised and used for the analysis of DNA 8-OHdG formation levels by HPLC-ECD, immunohistochemical assessment of cell proliferation (PCNA), and apoptosis (ssDNA). In the 78-week experiment (Exp. 2), animals were euthanized under the isoflurane when becoming moribund during the study or at the end of the experiment at week 78, autopsied, and the macroscopic pathological analysis was immediately performed. Mouse tissues and tumors were fixed in 10% buffered formalin and prepared for the routine histology (hematoxylin and eosin (H&E) staining) and immunohistochemistry or frozen in liquid nitrogen for proteome analysis.

### 2.4. Analysis of 8-OHdG Formation

Mouse liver DNA 8-OHdG levels were determined in experiment 1 after 4 weeks of PB treatment at a dose of 500 ppm by an HPLC-ECD method as described previously [32].

### 2.5. QSTAR Elite Hybrid LC-MS/MS

For QSTAR LC-MS/MS, samples from mouse liver HCCs, adenomas (HCAs), and surrounding and normal liver of PB-treated and control *Ogg1*^−/−^ and *Ogg1*^+/+^ mice from experiment 2 were prepared in 50 *μ*l of 9 M urea/2% CHAPS lysis buffer. Acetone precipitation was done to remove urea from the lysates. Then, reduction, alkylation, digestion with trypsin, and subsequent peptide labeling for each sample were performed using the AB Sciex iTRAQ Reagent Multi-Plex Kit as previously described [33, 34]. Proteome analysis was carried out using the QSTAR Elite Hybrid mass spectrometer (AB Sciex, Concord, ON, Canada) coupled to a DiNa-AI nano LC System (KYA Technologies, Tokyo, Japan). All reported data were used at 95% confidence cut-off limit. In quantitative analysis, protein lysates from frozen tumors (HCCs, adenomas) and normal-appearing areas from the liver of *Ogg1*^−/−^ and *Ogg1*^+/+^ mice treated with PB at a dose of 500 ppm and controls were digested and labeled with 4-plex iTRAQ reagents according to standard procedures [34, 35]. The pulled liver and tumor samples were labeled as follows. Set 1: 114, normal-appearing liver tissue from the control *Ogg1*^−/−^ male or female mice; 115, HCCs from PB-treated *Ogg1*^−/−^ male or female mice; 116, normal-appearing liver tissue from the control *Ogg1*^+/+^ male or female mice; 117, HCAs from PB-treated *Ogg1*^+/+^ male or female mice. Set 2: 114, surrounding liver tissue from PB-treated *Ogg1*^−/−^ male or female mice; 115, normal-appearing liver tissue from the control *Ogg1*^−/−^ male or female mice; 116, surrounding liver tissue from PB-treated *Ogg1*^+/+^ male or female mice; normal-appearing liver tissue from the control *Ogg1*^+/+^ male or female mice. Protein concentrations were measured with BCA Protein Assay Kit (Pierce, IL, USA).

### 2.6. Ingenuity Pathway Analysis (IPA)

We utilized the Ingenuity program (Ingenuity Systems, Mountain View, CA) to perform the functional, upstream regulators, pathway, and network analysis for interacting proteins (Ingenuity Systems, Mountain View, CA). Activation of upstream regulators or pathways was measured by *z*-scores. A *z*-score of above or less than 2 was considered significant.

### 2.7. Immunohistochemical Examination

In experiment 1, immunohistochemical staining for PCNA, a marker of cell proliferation, and apoptosis (single-stranded DNA (ssDNA)) was performed using an anti-PCNA rabbit polyclonal (PC-10, IgG; Santa Cruz Biotechnology Inc., Santa Cruz, CA; 1 : 500) and rabbit polyclonal ssDNA (IgG, 100 *μ*g/ml, Dako Japan Co., Kyoto, Japan; 1 : 400) antibodies, respectively, in liver sections as described previously using the ABC method [36]. The PCNA and ssDNA indices were estimated with counts of clearly brown/black positive nuclei per 1000 cells.

In experiment 2, differentially expressed proteins in HCCs and HCAs of mice treated PB were further verified by immunohistochemistry. Paraffin sections containing normal, surrounding liver tissue and tumors were used for comparison and stained using standard immunohistochemical methods. Guinea pig polyclonal antibodies against keratins 8 and 18 (KRT8/18) (1 : 400; Progen Biotechnik, Germany), rat monoclonal antibody against prohibitin 1 (PHB1) (1 : 300; Risk Assessment and Research Inc., Osaka, Japan), rabbit polyclonal antibody against cytochrome b5A (1 : 300; Santa Cruz Biotechnology Inc., California, USA), and rabbit monoclonal antibody against phospho-Nrf2 (Ser40) (1 : 100; Abcam, Tokyo, Japan) were employed. Antigen visualization was performed with 3,3′-diaminobenzidine tetrahydrochloride (Dako Japan). 8-OHdG and PCNA immunohistochemical evaluation was performed as described previously [36]. Different negative controls and antigen retrieval methods were used for optimization of the technique.

### 2.8. Western Blot Analysis

Livers and liver tumors of control or PB-treated *Ogg1*^−/−^ and *Ogg1*^+/+^ mice were lysed in T-PER Tissue Extraction Reagent (pH 7.6) (Pierce Biotechnology, Rockford, IL, USA), containing the proteinase inhibitor. Cell lysates were fractionated using the 10% SDS-PAGE and transferred to the Immobilon-P Transfer Membrane (Millipore, Billerica, MA). Membranes were incubated with primary antibodies and proteins visualized using an ECL Prime Western Blotting Detection Reagent according to the manufacturer's instructions (Amersham, Buckinghamshire, UK). Rabbit polyclonal antibodies against Nrf2 (ab31163; 1 : 1000) and rabbit monoclonal antibodies against p-Nrf2 (S40) (ab76026; 1 : 3000) were from Abcam (Tokyo, Japan) and mouse monoclonal antibody against *β*-actin (C4) (sc-47,778; 1 : 10,000) was from Santa Cruz Biotechnology (Santa Cruz, CA).

### 2.9. Statistical Analysis

The significance of differences between mean values was analyzed using the StatLight–2000(C) program (Yukms Corp., Japan). Kaplan-Meier analysis was conducted to determine the survival rates for *Ogg1^−/−^* and *Ogg1^+/+^* mice. The significance of intergroup differences in incidences of findings from gross pathology was analyzed by using Fisher's exact probability test (two sided). Statistical comparisons between the control and experimental groups and between the concomitant *Ogg1*^−/−^ and *Ogg1*^+/+^ groups for numerical data were assessed using the *F* test. If homogeneous, the data were analyzed with Student's *t*-test (two sided), and if not, with the Welch test.

## 3. Results

### 3.1. General Observations

No abnormalities in general condition of animals were found induced by PB treatment and no significant differences among the groups with regard to food and water consumption or body weight gain observed (data not shown). Total PB intake was comparable among *Ogg1* homozygous knockout and wild-type mice.

In experiment 2, significant increase in relative liver weights was found in the group of *Ogg1*^−/−^ (males: 6.3 ± 1.4 g, *P* < 0.0001; females: 6.2 ± 1.9 g, *P* < 0.01) and *Ogg1*^+/+^ (males: 8.3 ± 2.9 g, *P* < 0.0001; females: 6.8 ± 1.2 g, *P* < 0.0001) mice treated with PB as compared to corresponding control *Ogg1*^−/−^ (males: 4.2 ± 0.6 g; females: 4.9 ± 1.1 g) and *Ogg1*^+/+^ (males: 4.9 ± 1.0 g; females: 4.0 ± 1.3 g) animals. Trends for increase of relative spleen weights in PB-administered *Ogg1*^−/−^ (males: 0.23 ± 0.10 g; females: 0.36 ± 0.17 g) and *Ogg1*^+/+^ (males: 0.51 ± 0.45 g; females: 0.42 ± 0.23 g) mice as compared to corresponding control *Ogg1*^−/−^ (males: 0.16 ± 0.05; females: 0.29 ± 0.14 g) and *Ogg1*^+/+^ (males: 0.27 ± 0.15 g; females: 0.28 ± 0.10 g) mice were detected. Kidney weights did not differ between the PB-treated and control *Ogg1*^−/−^ or *Ogg1*^+/+^ groups.

### 3.2. Survival Curves (Exp. 2)

**Figure 1:**
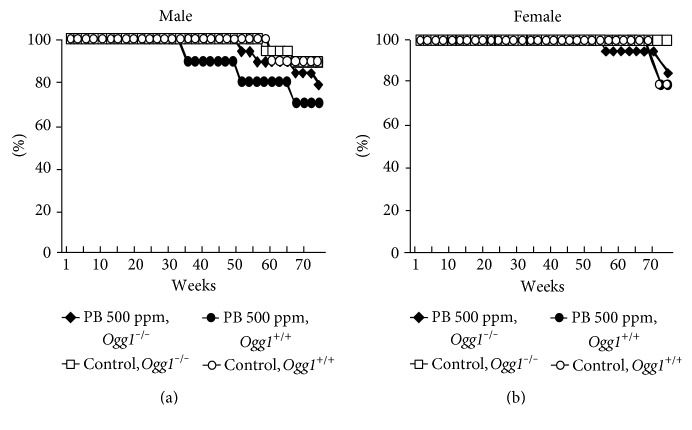
Survival curves for male (a) and female (b) *Ogg1*^−/−^ and *Ogg1*^+/+^ mice.

Survival curves for male and female *Ogg1* homozygous mutant and wild-type age-matched littermates in experiment 2 are shown in Figures 1(a) and 1(b), respectively. Nontreated female *Ogg1*^−/−^ mice appeared to be healthy and long-lived as compared to the corresponding control *Ogg1*^+/+^ mice. At week 38, the first PB-treated *Ogg1*^+/+^ male mouse with a malignant lymphoma was found. Spontaneous tumors, mostly malignant lymphomas/leukemias, developed in PB-administered *Ogg1*^+/+^ male mice were the reason of their earlier mortality. The number of PB-treated surviving *Ogg1*^−/−^ male and female mice started to decrease at weeks 52 and 55, respectively, mostly due to the development of malignant lymphomas/leukemias (females) and HCCs (males and females) (Figure 1()).

In experiment 2, no *Ogg1*^+/+^ animals died because of the development of liver tumors. In nontreated *Ogg1*^−/−^ male and female mice, the decrease of survival was observed at weeks 60 and 73, respectively (Figure 1).

### 3.3. Results of Histopathological Examination

Table 1 shows the incidence and general distribution of tumors in PB-treated and control male and female *Ogg1*^−/−^ and *Ogg1*^+/+^ mice genotypes.

PB-administered *Ogg1*^−/−^ mice were more susceptible to the induction of tumors as compared to *Ogg1*^−/−^ control littermates. PB treatment induced significant increase of total tumor incidence in *Ogg1*^−/−^ females (60%; *P* < 0.01), as compared to *Ogg1*^−/−^ control (10%); however, this effect was not observed in *Ogg1*^+/+^ mice (Table 1).

Neoplastic nodules induced in the PB-treated *Ogg1*^−/−^ mice were mainly malignant lymphomas/leukemias (females) and liver and lung tumors (Table 1). Histological examination of liver tumors at week 78 in experiment 2 demonstrated that all tumors developing in *Ogg1*^−/−^ animals administered PB were well-differentiated HCCs (10%), while PB-treated *Ogg1*^+/+^ animals developed only HCAs at the same rate (10%). Thus, in *Ogg1*^−/−^ mice, progression from HCA to HCC was obvious (Table 1). Malignant lymphomas/leukemias were found in PB-treated *Ogg1*^−/−^ females (35%), and males (5%), and differences were significant in females (*P* < 0.05) as compared to control *Ogg1*^−/−^ (0%) and PB-treated *Ogg1*^+/+^ (0%) mice. In nontreated homozygous mice, lymphomas/leukemias were completely absent, but observed in both sexes in control wild-type animals. Furthermore, lung adenomas were detected in both PB-treated and control *Ogg1*^−/−^ mice. Adenocarcinomas were found in the lungs of PB-administered *Ogg1*^−/−^ males, however, were absent in the PB-treated *Ogg1*^+/+^ group. In addition, uterine tumors (endometrial adenomas and sarcomas) were obvious only in PB-applied *Ogg1*^−/−^ and *Ogg1*^+/+^, but not in untreated *Ogg1*^−/−^ and *Ogg1*^+/+^ female mice.

### 3.4. 8-OHdG (HPLC-ECD) (Exp. 1)

**Figure 2:**
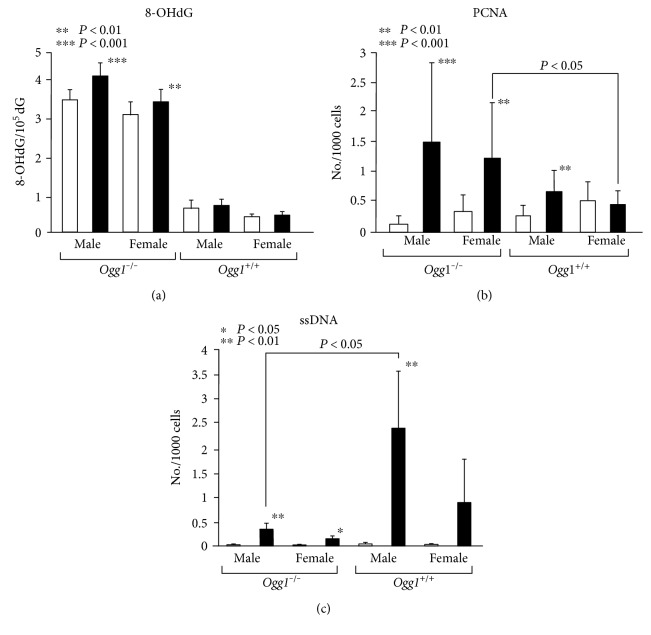
Formation of 8-OHdG and alterations to cell proliferation and apoptosis (ssDNA) in the livers of *Ogg1*^−/−^ and *Ogg1*^+/+^ mice treated with PB at 500 ppm for 4 weeks (opened squares: control groups; black squares: PB-treated groups).

The levels of 8-OHdG in the liver DNA of control and PB-treated *Ogg1* homozygous knockout mice were significantly higher than those observed in their wild-type counterparts (*P* < 0.0001) (Figure 2(a)). PB administration for 4 weeks resulted in comparable significant increases of 8-OHdG levels in male (*P* < 0.001) and female (*P* < 0.01) *Ogg1*^−/−^ mice as compared to the respective nontreated controls, but not in *Ogg1*^+/+^ animals, indicating that in the wild-type mice, 8-OHdG is successfully repaired by DNA repair enzymes (Figure 2(a)).

### 3.5. Alteration to Cellular Proliferation and Apoptosis (Exp. 1)

No significant differences of liver PCNA indices were observed between untreated *Ogg1*^−/−^ and *Ogg1*^+/+^ mice. In line with changes of DNA 8-OHdG formation, the significant induction of cell proliferation was found in the livers of male and female *Ogg1*^−/−^ mice administered PB for 4 weeks, and these values were higher than those observed in PB-treated *Ogg1*^+/+^ age-matched littermates (Figure 2(b)). Not high but still significant elevation of cell proliferation induced by PB was detected in the livers of male but not in female *Ogg1*^+/+^ mice as compared to *Ogg1*^+/+^ controls.

In experiment 1, the changes in apoptosis were controversial to that of PCNA (Figure 2(c)). Thus, PB application caused higher elevation of ssDNA positive cell indices in *Ogg1*^+/+^, as compared to the respective control. In *Ogg1*^−/−^ mice, small but still significant increase was found, because these values did not vary as much as it was observed with *Ogg1*^+/+^ mice (Figure 2(c)).

### 3.6. Alteration to Protein Expression Triggered by PB (Exp. 2)

The results of the QSTAR Elite MS/MS analysis of differentially expressed proteins in the livers and tumors of *Ogg1*^−/−^ and *Ogg1*^+/+^ mice, obtained after 78 weeks of PB administration in diet are presented in Table 2.

In both *Ogg1*^−/−^ and *Ogg1*^+/+^ PB-treated mice livers, comparable significant overexpression of CAR and PXR downstream enzymes involved in xenobiotic metabolism including CYP2B10, CYP3A11, CYP2A5, CYP1A2, CYP2C54, cytochrome b5 type A (CYB5A), carboxylesterase 1 (CES1), POR, GST alpha 3 (GSTA3) and alpha 4 (GSTA4), GST mu 1 (GSTM1), GST mu 3 (GSTM3), GST mu 5 (GSTM5), UDP-glucose 6-dehydrogenase (UGDH), and paraoxonase 1 (PON1) was demonstrated by proteome analysis. In addition, similar elevation of transferrin (TF), peroxiredoxin 1 (PRDX1) and 3 (PRDX3), and progesterone receptor membrane component 1 (PGRMC1) and downregulation of urea cycle enzymes such as arginase 1 (ARG1), carbamoyl-phosphate synthase 1 (CPS1), argininosuccinate lyase (ASL), ornithine carbamoyltransferase (OTC), and argininosuccinate synthase 1 (ASS1) in the surrounding liver tissue of both the *Ogg1*^−/−^ and *Ogg1*^+/+^ PB groups were found.

Interestingly, downstream proteins of Nrf2, for instance, GSTs, except GSTP1, were elevated in *Ogg1*^+/+^ HCAs, but showed only slight increase, no change, or were underexpressed in HCCs of *Ogg1*^−/−^ mice. Furthermore, elevation of Nrf2-related GSTM1, GSTM3, and GSTM5 in the surrounding liver tissue of *Ogg1*^+/+^ mice administered PB was higher than in the PB-treated *Ogg1*^−/−^ group. In addition, CYP2E1, the downstream proteins of *β*-catenin (CTNNB1) pathway, ubiquinol-cytochrome c reductase, Rieske iron–sulfur polypeptide 1 (UQCRFS1), and electron transfer flavoproteins *α* and *β*, involved in MTOR and RICTOR pathways were elevated only in HCCs of the *Ogg1*^−/−^ PB group. Furthermore, in *Ogg1*^−/−^ HCCs, significant overexpression of keratins 8 (KRT8) and 18 (KRT18), prohibitin 1 (PHB1), calreticulin (CALR), and histidine triad nucleotide-binding protein 1 (HINT1) transcriptional factors, vimentin (VIM), chitinase-like 3 (Chil3), serine dehydratase (SDS), methionine adenosyltransferase 1A (MAT1A), and PGRMC1 was detected. CYP2B10, CYP3A11, CYP2F2, and CES1, which are CAR and PXR downstream proteins, 4-hydroxyphenylpyruvate dioxygenase (HPD), glycine N-methyltransferase (GNMT), glycine-N-acyltransferase (GLYAT), paraoxonase 1 (PON1), and urea cycle enzymes were also overexpressed in PB-treated *Ogg1*^−/−^ HCCs, but not in *Ogg1*^+/+^ HCAs (Table 2). PPAR*α* downstream CYP2C54 and CYP2C70 were elevated in the livers and HCCs of PB-administered *Ogg1*^−/−^ mice. Furthermore, level of glutamate-ammonia ligase (glutamine synthetase (GLUL)), an enzyme involved in metabolism of glutamine and gamma aminobutyric acid (GABA), was increased in the livers, HCCs, and HCAs of both PB-treated *Ogg1*^−/−^ and *Ogg1*^+/+^ mice (Table 2). In the normal liver of control *Ogg1*^−/−^ animals, underexpression of several CAR, PXR, Nrf2, and PPAR*α* downstream proteins, as well as CALR and CANX was detected, likely being a result of adaptation to high levels of 8-OHdG in the DNA (Table 2).

Canonical pathways, protein functions, and upstream regulator analyses by IPA showed that PB treatment significantly altered the expression of proteins involved in Nrf2-mediated oxidative stress response in the surrounding liver and HCAs of *Ogg1*^+/+^ mice; however, in the liver and HCCs of the *Ogg1*^−/−^ group, Nrf2 activation level was low (Table 2 and Table S1 available online at https://doi.org/10.1155/2017/8541064). On the other hand, expression of CAR- and PXR-related proteins participated in the regulation of LPS/RXR, and xenobiotic metabolism were significantly increased in the livers of both PB-treated *Ogg1*^−/−^ and *Ogg1*^+/+^ animals. Furthermore, synthesis of reactive oxygen species and hydrogen peroxide was activated in *Ogg1*^−/−^ mouse HCCs, while inflammation and hepatic steatosis were predicted to be increased in the livers of *Ogg1*^−/−^ mice treated with PB (Table S2). Similarly, results of upstream regulator analysis indicated that CAR and PXR were significantly activated in the surrounding liver tissue of both *Ogg1*^−/−^ and *Ogg1*^+/+^ PB-treated animals. However, significant activation of Nrf2 transcriptional factor was predicted in surrounding livers and HCAs of *Ogg1*^+/+^ mice, but not in the livers or HCCs of the *Ogg1*^−/−^ group (Table S3). In addition, significant activation of *β*-catenin was predicted only in *Ogg1*^−/−^ mice HCCs, while RICTOR activity was increased in both *Ogg1*^+/+^ HCAs and *Ogg1*^−/−^ HCCs.

### 3.7. Immunohistochemical Evaluation

**Figure 3:**
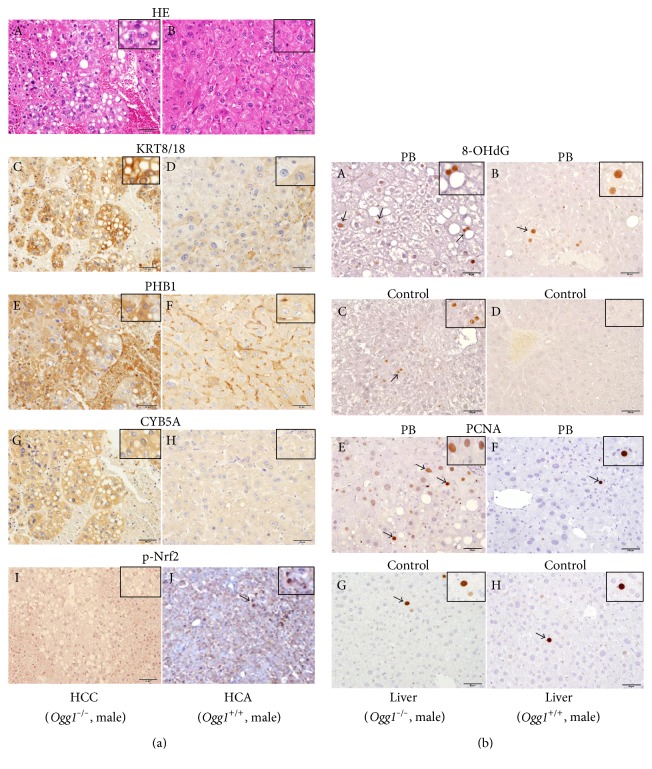
(a) Results of H&E staining and immunohistochemical evaluation of several hepatocarcinogenesis biomarkers (A–H) and p-Nrf2 (I, J) in the livers, HCC (A, C, E, G, and I), and HCA (B, D, F, H, and J) of *Ogg1*^−/−^ and *Ogg1*^+/+^ mice maintained on 500 ppm PB for 78 weeks. Note that the nuclear expression of p-Nrf2 in PB-treated *Ogg1*^+/+^ mice HCAs (arrow), but no staining in *Ogg1*^−/−^ HCCs. (b) Immunohistochemistry for 8-OHdG (A–D) and PCNA (E–H) in the surrounding liver tissue of *Ogg1*^−/−^ and *Ogg1*^+/+^ mice treated with PB for 78 weeks. Note the high expression of 8-OHdG (A) and PCNA (E) in the livers of *Ogg1*^−/−^ mice treated with PB (arrows).

Significant overexpression of KRT8/18, PHB1, and CYB5A in PB-treated *Ogg1*^−/−^ mouse HCCs and *Ogg1*^+/+^ HCAs was confirmed by the immunohistochemistry (Figure 3(a) (A–H)). Positive expression of phospho-Nrf2 (p-Nrf2) was observed in the cell nuclei and cytoplasm of HCAs of the PB-administered *Ogg1*^+/+^ group, while HCCs of *Ogg1*^−/−^ mice were negative (Figure 3(a) (I, J)).

In the surrounding liver tissue of *Ogg1*^−/−^ mice treated with PB for 78 weeks, increase of 8-OHdG positive expression in the hepatocyte nuclei as compared to the PB-treated *Ogg1*^+/+^ mice and nontreated control *Ogg1*^−/−^ mice was obvious (Figure 3(b) (A–D)). *Ogg1*^−/−^ control mice livers were characterized by high 8-OHdG expression, while *Ogg1*^+/+^ control mouse livers were completely negative. In line with 8-OHdG immunohistochemical results, similar staining pattern with highest expression level in PB administered *Ogg1*^−/−^ mice livers was observed for PCNA (Figure 3(b) (E–H)).

### 3.8. Lack of Nrf2 Activation in *Ogg1*^−/−^ Mice Livers and HCCs

**Figure 4:**
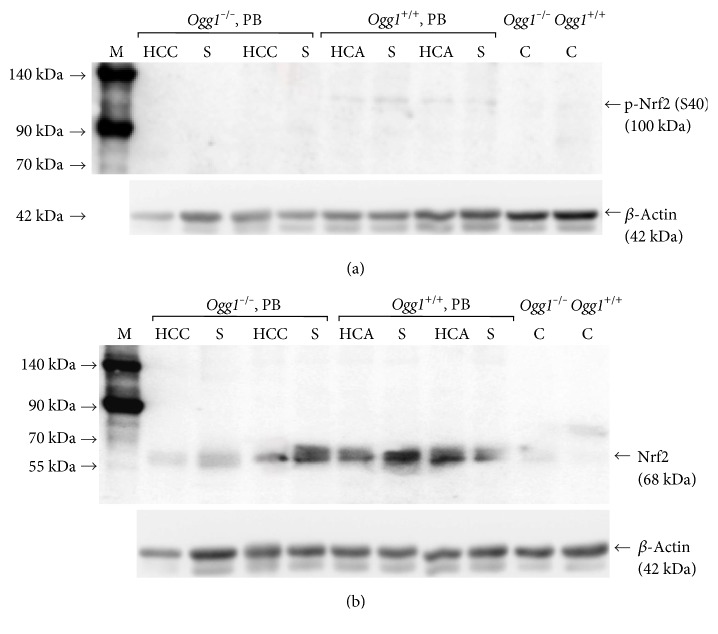
Representative blots for phospho-Nrf2 (p-Nrf2 (S40)) (a) and total Nrf2 (b) of *Ogg1*^−/−^ and *Ogg1*^+/+^ PB-treated and control mice are presented. PB induced elevation of total Nrf2 protein levels in the surrounding liver tissue and liver tumors of *Ogg1* null and mostly in the wild-type mice. However, p-Nrf2 was observed only in the livers and HCAs of wild-type animals, indicating that sufficient Nrf2 activation occurs only in PB-administered *Ogg1*^+/+^ mouse livers. HCC: hepatocellular carcinoma; HCA: hepatocellular adenoma; S: surrounding liver tissue; C: untreated control liver.

Western blot analysis demonstrated increase of Nrf2 phosphorylation in the livers and HCAs of PB-exposed wild-type mice and, on the contrary, lack of p-Nrf2 expression in the surrounding livers and HCCs of *Ogg1*^−/−^ mice (Figure 4(a)). However, overexpression of total Nrf2 protein was detected in both PB-treated *Ogg1*^−/−^ and *Ogg1*^+/+^ mice tumors and surrounding liver tissues.

## 4. Discussion

The present investigation revealed that long-term administration of nongenotoxic carcinogen PB, which is negative in the Ames test for mutagenicity, caused the progression of hepatocellular adenomas into carcinomas in homozygous *Ogg1* mutant mice deficient in 8-OHdG repair without the initiation treatment. In the wild-type C57BL/6J mice used as a background strain for *Ogg1* knockout, chronic PB application was previously reported to induce only HCAs but not HCCs [28]. Furthermore, PB has been further suggested to exert a dual role in liver tumor formation by promoting the growth of HCA but inhibiting the growth of HCC [37]. Thus, mutation and inactivation of *Ogg1* gene were associated with promotion of mouse hepatocarcinogenesis. Previously, inactivating mutations of DNA repair genes including *Ogg1* were reported in human liver, ovary, kidney, breast, and colon cancers [5, 38]. Furthermore, it was suggested that a positive correlation exists among human liver cancer stage, 8-OHdG levels, *Ogg1* polymorphisms, ALT/GGT levels, telomerase activity, and overexpression of miR-92, a microRNA that plays a role in both the apoptotic process and the cellular proliferation pathways [39]. Our results supported the idea that increase of oxidative base modifications in case of the defective DNA repair is resulted in development of HCC in mice.

Untreated *Ogg1* homozygous mutant mice developed spontaneously tumors only in the lungs, which might be due to the significant accumulation of nonrepaired oxidative DNA base modifications in this organ, which is strongly exposed to the molecular oxygen. These data are in line with those reported by Xie et al. [19] and Sakumi et al. [15], demonstrating the spontaneously developed lymphomas, lung and ovary tumors by *Ogg1*^−/−^ mice. In our study, untreated homozygous mutant, mostly female mice were found to be healthy and long-lived as compared to their wild-type counterparts, in spite of very high level of 8-OHdG in the tissues, indicating that these animals have an adaptation system to high levels of oxidative DNA damage produced due to the knockout of *Ogg1* gene. Thus, no lymphomas/leukemias were found in nontreated *Ogg1*^−/−^ mice, therefore, affecting their survival. In the liver of control homozygous mutant mice, one of the possible adaptive mechanisms to high levels of oxidative DNA damage could be related to the downregulation of several CAR-, PXR-, Nrf2-, and PPAR*α*-related proteins, CALR, and CANX. We further observed that PB-treated female homozygous mice were highly susceptible to the development of malignant lymphomas/leukemias, likely being indicative of existing gender differences in response to PB treatment.

**Figure 5:**
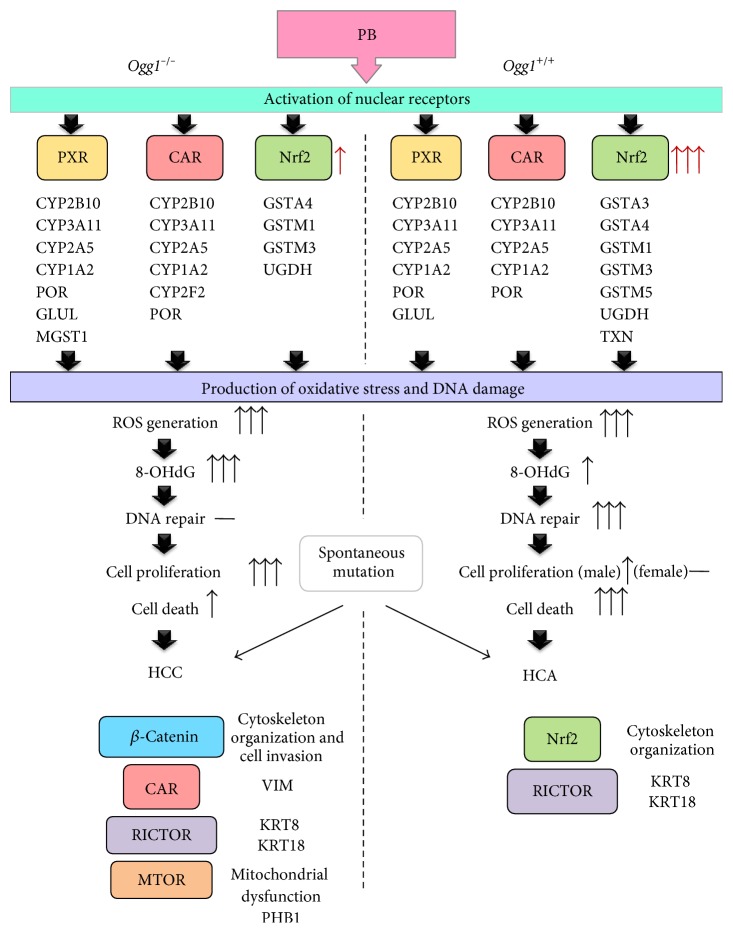
Flow chart on mechanisms of hepatocarcinogenesis in the *Ogg1*^−/−^ and *Ogg1*^+/+^ mice. ↑: activation, overexpression, elevation; −: no change.

It has been demonstrated that PB-induced liver-specific DNA damage in mice can be attributed to free radicals, particularly hydroxyl or superoxide radicals arising from the induction of phase I metabolizing enzymes such as cytochrome P450 by PB [36, 40, 41]. The present observation of significant 8-OHdG elevation in the liver DNA induced by PB in *Ogg1*^−/−^ mice is in line with our previous data obtained in rats with regard to promotion of liver carcinogenesis [22]. Furthermore, depletion in cellular glutathione may be correlated with reactive oxygen species mediated oxidative stress [42]. From the results of the proteome analysis, PB application caused significant CAR- and PXR-dependent induction of phase I and II metabolic enzymes in both *Ogg1* homozygous mutant and wild-type mice, but insufficient, or no Nrf2 activation in the *Ogg1*^−/−^ livers and HCCs, respectively, pointing out that nonrepaired 8-OHdG and uncontrolled accumulation and damage from the reactive oxygen species in *Ogg1*^−/−^ liver tissue could become the reason of progression of hepatic adenoma to carcinoma (Figure 5()).

In previous studies, mice that lack the Nrf2 transcription factor were more sensitive to the cytotoxic and genotoxic effects of foreign chemicals and oxidants than wild-type animals [43]. In line with our data, Nrf2 ablation has been previously shown to suppress GSTA1, GSTM1, GSTM3, GSTM4, and PRDX1 [43, 44]. Furthermore, Nrf2 has been shown to upregulate the activity of multiple DNA repair pathways, including a pathway involved in the removal of oxidative stress-induced endogenous DNA interstrand crosslinks. In our study, 8-OHdG formation levels were significantly lower in the livers of *Ogg1*^+/+^ mice; therefore, they were more resistant to PB treatment in comparison with *Ogg1*^−/−^ due to active DNA repair and Nrf2. It could be suggested that accumulation of unrepaired 8-OHdG in the livers of PB-treated *Ogg1*^−/−^ animals caused a significant increase of cellular proliferation. Our findings point out the existence of interrelation between the base excision repair for oxidative DNA modifications, Nrf2 signaling pathway, and cell proliferation. The defective 8-OHdG repair was accompanied by lack of Nrf2 phosphorylation, what likely contributed to progression of hepatocarcinogenesis in *Ogg1*^−/−^ mice induced by PB. Coordinated overexpression of Ogg1 and Nrf2 and downregulation in Keap1 expression were previously shown in HepG2 human liver cancer cell line after the menadione and H_2_O_2_/Fe^2+^ exposure [45]. Furthermore, level of Ogg1 and nuclear translocation of Nrf2 protein were reported to be correlatively decreased upon treatment with PI3K or Akt inhibitors, indicating the existence of crosstalk between Ogg1 and Nrf2 [46]. Furthermore, increase of Nrf2 expression but not its activity was previously shown in human liver cancer samples [47]. However, increase of Nrf2 expression, which could be compensatory, does not necessarily mean elevation of its activity, which was reported to be increased due to phosphorylation by oxidative stress-activated different kinases such as ERK, JNK, PKC, and PI3K/AKT, and leads to dissociation of Nrf2 from Keap1 with subsequent nuclear transportation [48]. From our results, in contrast to the wild-type mice, in the livers of *Ogg1*-deficient animals, Nrf2 phosphorylation and likely its transformation to the nucleus did not occur, resulting in significant increase of oxidative stress and DNA damage of liver cells, and finally, development of HCC.

Previously, an increase in mutation frequency has been shown to be induced by the administration of potassium bromate in *Ogg1*^−/−^ mouse liver after partial hepatectomy [21]. This study suggested that high levels of cell proliferation are extremely important for the fixation of mutations induced by oxidative stress conditions in the liver. From our results, in *Ogg1*^+/+^, but not *Ogg1*^−/−^ mice, PB further caused significant elevation of p53-associated apoptosis in the surrounding liver tissue. Previously, we have demonstrated that in about 70% of cells with DNA-damaged nuclei in the rat liver induced by PB are apoptotic, suggesting that significant elevation induces DNA fragmentation [36]. Thus, the mechanism of PB carcinogenicity in the liver of *Ogg1*^−/−^ mice could be related to the accumulation of nonrepaired oxidative DNA modifications leading to mutations, elevation of cell proliferation, and suppression of apoptosis what likely resulted in progression of hepatocarcinogenesis.

Significant elevation of proteins inducible by oxidative stress, which could participate in progression of HCA to HCC, such as KRT8, KRT18, PHB1, CALR, and VIM, was observed in the *Ogg1*^−/−^ mouse HCCs. In line with the present results, we have previously reported overexpression of KRT8/18 and PHB1 as biomarkers of mouse liver preneoplastic lesions and tumors, showing the highest expression in carcinomas [34, 35, 49]. In addition, here, we observed significant increase of GLUL expression in the livers and tumors of both PB-treated *Ogg1*^−/−^ and *Ogg1*^+/+^ animals. GLUL is present in the brain and liver and is involved in nitrogen homeostasis [50]. Christa et al. reported that GLUL is overexpressed in primary liver cancers, indicating its potential role in the transformation of hepatocytes [50].

In the present study, HCCs developed in PB-treated *Ogg1* homozygous mutant mice were further characterized by activation of Wnt/*β*-catenin signaling in association with activation of RICTOR and slight activation of MTOR and CAR signaling. Previously, activation of *β*-catenin in human and mouse HCCs have been demonstrated [51, 52]. In line with our data, single *β*-catenin activation has been reported to be insufficient for induction of liver cancer in mice, while combining CAR and *β*-catenin activation resulted in tumorigenesis [53].

## 5. Conclusions

The present results indicate that accumulation of reactive oxygen species and unrepaired oxidative DNA damage produced by PB is associated with suppression of Nrf2 pathway and significant elevation of cellular proliferation in the livers of *Mmh/Ogg1*-deficient mice, likely being related to progression from hepatocellular adenoma to carcinoma.

## Supplementary Material

Table S1. Canonical pathways analysis by IPA in the livers and liver tumors of control and PB-treated Ogg1−/− and Ogg1+/+ mice. Table S2. Altered biofunctions in the livers and liver tumors of control and PB-treated Ogg1−/− and Ogg1+/+ mice. Table S3. Upstream regulator analysis by IPA in the livers and liver tumors of control and PB-treated Ogg1−/− and Ogg1+/+ mice.

## Figures and Tables

**Table 1:** Results of the histopathological analysis in *Ogg1*^−/−^ and *Ogg1*^+/+^ mice.

	PB, 500 ppm		Control	
Gender	Male	Female	Male	Female
*Ogg1* ^−/**−**^				
Number of animals	20	20	20	20
Number of tumor-bearing mice (%)	5 (25)	12 (60)^a^	3 (15%)^∗^	2 (10%)^∗^
Number of tumors/mouse	0.25	0.6^a^	0.15^∗∗^	0.1^∗∗^
*Ogg1* ^+/+^				
Number of animals	20	20	20	20
Number of tumor-bearing mice (%)	10 (50)	10 (50)	10 (50)	10 (50)
Number of tumors/mouse	0.5	0.5	0.5	0.5
*Ogg1* ^−/**−**^				
M. lymphoma/leukemia	1 (5)	7 (35)^a,^^∗∗^	0	0
Liver				
HCA	0	0	0	0
HCC	2 (10)	2 (10)	0	0
Lung				
Adenoma	1 (5)	2 (10)	3 (15)	2 (10)
Adenocarcinoma	2 (10)	0	0	0
Uterus				
Endometrial adenoma	—	1 (5)	—	0
Tumor incidence (%)^b^				
*Ogg1* ^+/+^				
M. lymphoma/leukemia	6 (30)	0	2 (10)	4 (20)
Liver				
HCA	2 (10)	2 (10)	0	0
HCC	0	0	0	0
Lung				
Adenoma	2 (10)	2 (10)	0	2 (10)
Adenocarcinoma	0	0	0	0
Uterus				
Endometrial adenoma	—	2 (10)	—	0
Sarcoma	—	2 (10)	—	0
Renal				
Adenocarcinoma	0	0	2 (10)	0
Pituitary gland				
Adenoma	0	2 (10)	0	2 (10)
Zymbal gland				
Adenoma	0	0	0	2 (10)
Subcutis				
Fibrosarcoma	2 (10)	0/0	4 (20)	0
Pancreas				
Adenoma	0	0	2 (10)	0
Ductal adenocarcinoma	0	2 (10)	0	0

^a^ *P* < 0.01 versus the *Ogg1*^−/−^ control group; ^∗^*P* < 0.05 and ^∗∗^*P* < 0.01 versus the control or PB-treated *Ogg1*^+/+^ group. ^b^Only organs with neoplastic lesions are listed.

**Table 2:** Differentially expressed proteins identified by LC-MS/MS in the liver tumors, surrounding and normal-appearing liver tissue of *Ogg1*^−/−^ and *Ogg1*^+/+^ mice treated with PB for 78 weeks.

Protein name (symbol)	GI number	*Ogg1* ^−/−^ versus *Ogg1*^+/+^ liver		PB, *Ogg1*^−/−^ liver		PB, *Ogg1*^+/+^ liver		*Ogg1* ^−/−^ HCC		*Ogg1* ^+/+^ HCA			
		Ratio	*P* value	Ratio	*P* value	Ratio	*P* value	Ratio	*P* value	Ratio	*P* value	Location	Function(s)/upstream regulator
Cytochrome P450 fam. 2 subfam. E mem. 1 (CYP2E1)	461828	-	-	-	-	-	-	3.26	0.00	1.54	0.00	EPR	XM/CTNNB1
Cytochrome b5 type A (CYB5A)	3023608	-	-	1.65	0.00	1.64	0.00	1.41	0.00	1.43	0.00	EPR	FAM/CTNNB1
Cytochrome P450 fam. 1 subfam. A mem. 2 (CYP1A2)	117146	0.87	0.03	2.79	0.00	2.03	0.00	1.40	0.01	2.36	0.00	EPR	XM/PXR, CAR
Cytochrome P450 fam. 2 subfam. F mem. 2 (CYP2F2)	461829	0.89	0.03	1.23	0.03	-	-	2.04	0.01	0.14	0.02	EPR	XM, SM/CAR
Cytochrome P450 fam. 2 subfam. A mem. 5 (CYP2A5)	117196	1.42	0.00	2.57	0.00	3.23	0.00	1.68	0.00	1.84	0.00	EPR	XM/PXR, CAR
Cytochrome P450 fam. 2 subfam. B mem. 10 (CYP2B10)	117215	-	-	3.78	0.00	4.58	0.01	2.48	0.00	-	-	EPR	XM/CAR, PXR
Cytochrome P450 fam. 3 subfam. A mem. 11 (CYP3A11)	5921911	-	-	2.06	0.00	2.08	0.00	2.59	0.02	-	-	EPR	XM/PXR, CAR
Carboxylesterase 1 (CES1)	57013389	1.20	0.00	1.26	0.00	1.24	0.00	2.90	0.00	0.85	0.00	EPR	XM, TgM/PXR
Glutamate-ammonia ligase, glutamine synthetase (GLUL)	145559476	0.80	0.00	1.60	0.04	2.81	0.01	2.72	0.00	2.39	0.00	C	GABAM/PXR
Transferrin (TF)	21363012	-	-	1.23	0.00	1.38	0.00	0.67	0.00	1.26	0.00	ES	CIIH, AFO/PXR
Sulfotransferase fam. 1A. mem. 1 (Sult1a1)	1711570	1.28	0.00	0.69	0.00	1.20	0.02	2.48	0.00	1.56	0.00	C	XM, SM/CAR
Cytochrome P450 oxidoreductase (POR)	548338	1.20	0.00	1.38	0.00	1.92	0.00	1.76	0.00	1.29	0.00	EPR	CRH/PXR, CAR
Superoxide dismutase 1, soluble (SOD1)	134614	-	-	0.93	0.02	0.92	0.04	1.20	0.00	1.31	0.00	C	CRH/Nrf2
Thioredoxin (TXN)	549078	-	-	-	-	1.22	0.03	-	-	1.39	0.05	C	CRH/Nrf2
Peroxiredoxin 1 (PRDX1)	547923	0.85	0.01	1.20	0.00	1.25	0.00	1.25	0.00	2.19	0.00	C	CRH/Nrf2
Glutathione S-transferase, alpha 3 (GSTA3)	232203	-	-	-	-	1.23	0.01	1.26	0.01	2.53	0.00	C	GM/Nrf2, PXR, CAR
Glutathione S-transferase, alpha 4 (GSTA4)	20141353	-	-	1.73	0.01	1.61	0.02	0.68	0.01	2.05	0.00	O	GM/Nrf2, PXR, CAR
Glutathione S-transferase, mu 1 (GSTM1)	121718	-	-	2.31	0.00	2.70	0.00	1.36	0.01	4.40	0.00	Mit	GM/Nrf2, PXR, CAR
Glutathione S-transferase, mu 3 (GSTM3)	121720	-	-	3.99	0.00	6.08	0.00	1.38	0.01	3.61	0.00	C	GM/Nrf2, PXR, CAR
Glutathione S-transferase, mu 5 (GSTM5)	121716	-	-	2.59	0.00	3.07	0.00	-	-	2.06	0.00	C	GM/Nrf2, PXR, CAR
Microsomal glutathione S-transferase 1 (MGST1)	47116030	0.72	0.00	1.20	0.05	-	-	1.50	0.00	0.73	0.01	EPR	GM/PXR
Glutathione S-transferase, pi 1 (GSTP1)	121747	0.76	0.00	-	-	0.64	0.00	0.71	0.00	0.69	0.00	C	GM/Nrf2
UDP-glucose 6-dehydrogenase (UGDH)	6136117	-	-	1.74	0.02	1.70	0.02	0.69	0.00	1.68	0.00	N	CM/Nrf2
Arginase 1 (ARG1)	2492934	-	-	0.71	0.00	0.81	0.00	1.39	0.00	0.15	0.00	C	M, UC
Carbamoyl-phosphate synthase 1 (CPS1)	73918911	0.96	0.01	0.75	0.00	0.77	0.00	1.40	0.00	0.19	0.00	C	M, UC
Argininosuccinate lyase (ASL)	21263402	-	-	0.81	0.00	0.88	0.02	1.55	0.00	0.23	0.00	C	M, UC
Ornithine carbamoyltransferase (OTC)	129277	0.91	0.04	0.89	0.01	0.77	0.00	1.24	0.00	0.11	0.00	C	M, UC
Argininosuccinate synthase 1 (ASS1)	114290	0.84	0.00	0.72	0.00	0.78	0.00	2.45	0.00	0.14	0.00	C	M, UC
Cytochrome P450, fam. 2, subfam. c, mem. 54 (CYP2C54)	81893400	-	-	2.90	0.00	1.99	0.00	4.32	0.03	0.56	0.02	EPR	XM, FAM/PPARA
Cytochrome P450, fam. 2, subfam. c, mem. 70 (CYP2C70)	83288029	0.71	0.03	1.33	0.01	-	-	2.06	0.03	0.73	0.00	EPR	XM, SM/PPARA
Cytochrome c oxidase subunit II (MT-CO2)	117029	-	-	-	-	-	-	1.28	0.00	0.30	0.00	Mit	CRH, FABO/PPARA
4-Hydroxyphenylpyruvate dioxygenase (HPD)	83303597	0.88	0.01	-	-	-	-	2.05	0.00	0.64	0.00	C	TyrM/PPARA
Glycine N-methyltransferase (GNMT)	55976615	0.86	0.00	0.75	0.00	0.89	0.00	2.19	0.00	0.31	0.00	C	GlyM/PPARA
Glycine-N-acyltransferase (GLYAT)	81879668	-	-	-	-	0.87	0.00	1.29	0.01	0.79	0.02	C	GlyM
Indolethylamine N-methyltransferase (INMT)	731019	-	-	1.33	0.00	0.77	0.03	1.80	0.00	0.31	0.03	C	AM
Paraoxonase 1 (PON1)	1709718	-	-	1.77	0.00	2.00	0.01	7.38	0.00	-	-	ES	XM, ChM/PPARG
Vimentin (VIM)	138536	-	-	1.42	0.05	-	-	1.66	0.01	-	-	C	IFO, A(−)
Phosphoenolpyruvate carboxykinase 1 (PCK1)	18203662	-	-	0.60	0.02	-	-	2.63	0.03	-	-	C	CM/PPARG
Peroxiredoxin 2 (PRDX2)	2499469	-	-	-	-	-	-	0.73	0.01	-	-	C	CRH/ERBB2. MYC
Peroxiredoxin 3 (PRDX3)	126986	-	-	1.16	0.03	1.13	0.05	0.59	0.00	2.36	0.00	Mit	CRH
Peroxiredoxin 5 (PRDX5)	20141789	0.87	0.04	-	-	0.88	0.02	0.78	0.01	-	-	Mit	CRH
Peroxiredoxin 6 (PRDX6)	3219774	-	-	-	-	-	-	0.80	0.00	1.75	0.00	L	CRH, LC
Ubiquinol-cytochrome c reductase, Rieske iron–sulfur polypeptide 1 (UQCRFS1)	52000877	-	-	-	-	-	-	1.33	0.04	-	-	Mit	XM/RICTOR
Electron transfer flavoprotein alpha subunit (ETFA)	146345417	-	-	0.79	0.00	0.78	0.00	1.32	0.00	-	-	Mit	FABO/MTOR
Electron transfer flavoprotein beta subunit (ETFB)	92090596	-	-	0.82	0.00	0.78	0.00	1.22	0.00	-	-	Mit	FABO/ERBB2, MTOR
Methionine adenosyltransferase 1A (MAT1A)	81902386	-	-	0.63	0.00	1.14	0.01	2.99	0.00	0.40	0.00	C	MC/SREBF1
Aminoadipate-semialdehyde synthase (AASS)	46395955	-	-	0.70	0.00	-	-	2.80	0.00	0.43	0.00	C	LysD/CREB
3-Hydroxyisobutyrate dehydrogenase (HIBADH)	32363159	-	-	0.85	0.01	-	-	2.30	0.01	-	-	C	VC/MYC, FOXO4
Serine dehydratase (SDS)	52783414	-	-	-	-	-	-	2.09	0.02	0.05	0.01	C	CM/FOXA2
Chitinase-like 3 (Chil3)	51315803	2.52	0.01	0.41	0.01	-	-	3.59	0.00	-	-	C	CM/IL4
Keratin 18 (KRT18)	148886614	-	-	-	-	1.34	0.00	2.55	0.00	1.49	0.00	C	IFO, A(−)/TGFB1
Keratin 8 (KRT8)	90110028	-	-	-	-	1.33	0.00	2.60	0.00	1.44	0.00	C	IFO, A(−)/TGFB1
Vitronectin (VTN)	1722806	-	-	-	-	9.54	0.02	-	-	-	-	ES	CMA/FXR, LXR
Serum amyloid A1 (SAA1)	134159	-	-	1.20	0.04	30.47	0.00	-	-	-	-	ES	APR/FXR, LXR
Prohibitin 1 (PHB1)	54038835	-	-	0.81	0.02	-	-	1.47	0.01	-	-	Mit	TR, MitO/IL6, MYC
Histidine triad nucleotide-binding protein 1 (HINT1)	2495231	-	-	-	-	-	-	1.29	0.05	-	-	N	TR, PurC/TGFB1
Calreticulin (CALR)	117502	0.91	0.01	-	-	-	-	1.22	0.01	-	-	EPR	TR, CP(+)/Ca2+
Calnexin (CANX)	543921	0.88	0.03	-	-	-	-	1.40	0.00	-	-	EPR	PF/MYC, EGF
Cathepsin D (CTSD)	115718	-	-	-	-	1.30	0.01	2.37	0.00	-	-	L	Pro/TGFB1
Nipsnap homolog 1 (*C. elegans*) (NIPSNAP1)	17380130	-	-	-	-	-	-	1.44	0.01	-	-	Mit	NB/FOS, YY1
Progesterone receptor membrane comp. 1 (PGRMC1)	46577676	-	-	1.39	0.03	1.43	0.00	2.61	0.01	-	-	PM	SB/MTOR, TGFB1
Receptor accessory protein 6 (REEP6)	81881956	-	-	-	-	-	-	1.71	0.04	0.65	0.03	PM	ITR/RET

C: cytoplasm; EPR, endoplasmic reticulum; ES: extracellular space; L, lysosome; M, mitochondria; N: nucleus; PM: plasma membrane; A(−): negative regulation of apoptosis; AFO: actin filament organization; AM: amine metabolism; APR: acute phase response; ChM: cholesterol metabolism; CIIH: cellular iron ion homeostasis; CM: carbohydrate metabolism; CMA: cell-matrix adhesion: CRH: cell-redox homeostasis: CP(+): positive regulation of cell proliferation; CR(−): negative regulation of coagulation; GABAM: GABA metabolism; GlyM: glycine metabolism; GM: glutathione metabolism; ITR: intracellular transport regulation; FABO: fatty acid beta oxidation; FAM: fatty acid metabolism; IFO: intermediate filament organization; LC: lipid catabolism; LysD: lysine degradation; M: metabolism; MC: methionine catabolism; MCF: mitochondria cristae formation; MitO: mitochondrial organization; NB: neurotransmitter binding; NM: nucleotide metabolism; Pro: proteolysis; PurC: purine ribonucleotide catabolism; SB: steroid binding; SM: steroid metabolism; TCA: TCA cycle; TgM: triglyceride metabolism; TR: transcription regulation; TyrM: tyrosine metabolism; VC: valine catabolism; XM: xenobiotic metabolism; UC: urea cycle; “-”: protein expression is not changed; “0.000, *P* < 0.0001.”
